# Co-expression of cancer driver genes: IDH-wildtype glioblastoma-derived tumorspheres

**DOI:** 10.1186/s12967-020-02647-8

**Published:** 2020-12-14

**Authors:** Seon-Jin Yoon, Hye Young Son, Jin-Kyoung Shim, Ju Hyung Moon, Eui-Hyun Kim, Jong Hee Chang, Wan Yee Teo, Se Hoon Kim, Sahng Wook Park, Yong-Min Huh, Seok-Gu Kang

**Affiliations:** 1 Department of Biochemistry and Molecular Biology, College of Medicine, Yonsei University, Seoul, Korea; 2 Brain Korea 21 PLUS Project for Medical Science, Yonsei University, Seoul, Korea; 3 Severance Biomedical Science Institute, College of Medicine, Yonsei University, Seoul, Korea; 4 Department of Neurosurgery, Brain Tumor Center, Severance Hospital, College of Medicine, Yonsei University, 50-1 Yonsei-ro, Seodaemun-gu, Seoul, 03722 Republic of Korea; 5 Cancer and Stem Cell Biology Program, Duke-NUS Medical School, Singapore, Singapore; 6 National Cancer Center, Singapore, Singapore; 7 KK Women’s and Children’s Hospital, Singapore, Singapore; 8 Institute of Molecular and Cell Biology, A*STAR, Singapore, Singapore; 9 Department of Pathology, Severance Hospital, College of Medicine, Yonsei University, Seoul, Korea; 10 Department of Radiology, Severance Hospital, Yonsei University College of Medicine, 50-1 Yonsei-ro, Seodaemun-gu, Seoul, 03722 Republic of Korea; 11 YUHS-KRIBB Medical Convergence Research Institute, Seoul, Republic of Korea; 12 Department of Medical Science, Yonsei University Graduate School, Seoul, Korea

**Keywords:** Isocitrate dehydrogenase-wildtype glioblastoma, Transcriptome, Single cell RNAseq, Tumorsphere

## Abstract

**Background:**

Driver genes of GBM may be crucial for the onset of isocitrate dehydrogenase (*IDH*)-wildtype (WT) glioblastoma (GBM). However, it is still unknown whether the genes are expressed in the identical cluster of cells. Here, we have examined the gene expression patterns of GBM tissues and patient-derived tumorspheres (TSs) and aimed to find a progression-related gene.

**Methods:**

We retrospectively collected primary *IDH*-WT GBM tissue samples (*n* = 58) and tumor-free cortical tissue samples (control, *n* = 20). TSs are isolated from the *IDH*-WT GBM tissue with B27 neurobasal medium. Associations among the driver genes were explored in the bulk tissue, bulk cell, and a single cell RNAsequencing techniques (scRNAseq) considering the alteration status of *TP53*, *PTEN*, *EGFR*, and *TERT* promoter as well as *MGMT* promoter methylation. Transcriptomic perturbation by temozolomide (TMZ) was examined in the two TSs.

**Results:**

We comprehensively compared the gene expression of the known driver genes as well as *MGMT*, *PTPRZ1*, or *IDH1*. Bulk RNAseq databases of the primary GBM tissue revealed a significant association between *TERT* and *TP53* (p < 0.001, R = 0.28) and its association increased in the recurrent tumor (p  < 0.001, R = 0.86). TSs reflected the tissue-level patterns of association between the two genes (p < 0.01, R = 0.59, n = 20). A scRNAseq data of a TS revealed the *TERT* and *TP53* expressing cells are in a same single cell cluster. The driver-enriched cluster dominantly expressed the glioma-associated long noncoding RNAs. Most of the driver-associated genes were downregulated after TMZ except *IGFBP5*.

**Conclusions:**

GBM tissue level expression patterns of *EGFR*, *TERT*, *PTEN*, *IDH1*, *PTPRZ1*, and *MGMT* are observed in the GBM TSs. The driver gene-associated cluster of the GBM single cells were enriched with the glioma-associated long noncoding RNAs.

## Background

Glioblastoma (GBM) has been known as the heterogeneous tumor with necrotic portion, perivascular proliferation, or its infiltrative nature to the surrounding cortex [1, 2]. As the diagnostic tissue slides show these molecularly different areas, cellular models of GBM have been questioned for its reliability [3, 4].

Molecular subtypes have been proposed to account for the heterogeneity of GBM [1, 5]. However, these subtypes are not used in clinical diagnosis because of their stochastic nature [6]. To increase the accuracy of diagnosis, clinical glioma classification has recently gravitated toward the analysis of mutations on the core driver genes, such as those of isocitrate dehydrogenase (*IDH*), epidermal growth factor receptor (*EGFR*), phosphatase and tensin homolog (*PTEN*), tumor protein p53 (*TP53*), telomerase reverse transcriptase (*TERT*) promoter, protein tyrosine phosphatase receptor type Z1 (*PTPRZ1*), as well as *O*-6-methylguanine-DNA methyltransferase (*MGMT*) promoter methylation [2, 7].

*IDH* is the primary gene used to distinguish between primary and secondary GBM [8]. Their clinical incidence and molecular evidence suggest that these tumor types differ in their mutation and expression profiles [5, 9, 10]. However, despite its importance, approximately 80% of patients with GBM patients have *IDH*-wildtype (WT) tumors [11, 12]. Furthermore, most of the established PDX models are from *IDH*-WT GBM which may suggest its importance in the survival of cells [13–15].

*EGFR*, *PTEN*, and *TP53* mutation are the most common mutation in the GBM [16, 17]. Among these driver genes, *TP53* mutation shows biased distribution when grouped by IDH-mutation status: *IDH*-mutant GBM with 75% of mutations while *IDH*-WT GBM with 26.7% *TP53* mutant cases [2]. Even though, gain-of-function phenotype by *TP53* mutation suggests the harmful effect of *TP53* mutation [18–24], its prognostic impact is still controversial in the GBM and other cancers [22, 25, 26].

*TERT* activity is detected in up to 90% of human primary cancer [27]. The rate of *TERT* promoter mutations is reported as about 58–90% of *IDH*-WT GBM patients [27, 28]. And 94% of GBM cells are reported to harbor *TERT* mutation [29]. However, *TERT* promoter mutation does not significantly affect the prognosis of GBM patients [28].

*PTPRZ1* shows a relatively low rate of mutation in the GBM. Recently, this gene is being associated with the origin of glioma cells with the elevated expression in the GBM tissue as well as the subventricular zone [30, 31]. As a marker of neuroglial origin, PTPRZ1 may add a bridge between the neurotransmitters, neurodevelopment, and tumor microtubes [10, 30, 32, 33].

*MGMT* promoter methylation status is observed in the 50% of glioblastoma patients. Its promoter methylation status is correlated with the gene expression [34]. In GBM, unmethylated *MGMT* promoter status is associated with poor response to alkylating agents [35, 36]. Temozolomide (TMZ) is the most important alkylating agent available in the GBM patients [37]. However, contrasting reports shows other mechanisms than *MGMT* promoter methylation may be involved in the *MGMT*-deficient GBM cells [38].

Here, a retrospective comparative analysis of RNAseq and single cell RNAseq data from *IDH*-WT GBM and GBM TS was conducted to find whether the TSs are representing the signatures of tumor tissue. Furthermore, we aimed to find the transcriptomic change after TMZ treatment in the in vitro level.

## Methods

### Clinical samples

*IDH*-WT GBM tissue samples were obtained from Brain cancer center, Severance hospital (*n* = 58, from 2016 to 2020, The patient samples were ethically approved by the institutional review board of Severance hospital). Tumor-free cortex samples for control were obtained when available during the resection of subcortical tumors, *n* = 24). All samples with associated DNA mutation profiles and tumor RNAseq data were included in this retrospective analysis. Samples without tumor mutation profiles were included for comparison. Clinical information, Mutation profiles, and *MGMT* promoter methylation status were obtained from the electronic medical record of the hospital. Detailed methods are described in each section. Mutation profiles were not evaluated for the healthy cortex controls, but were extrapolated from the results of the matched tumor tissues. Frozen tissue samples of RNAseq were processed in the (Theragen, Seongnam-si, Republic of Korea).

### Tumorsphere culture

Patient samples of *IDH*-WT glioblastoma were cultured with the neurosphere media within 1 h after surgical resection [39–45]. Patient-derived TSs were established from the fresh GBM tissue specimens as previously described (*n* = 23, Institutional review board review number, 2012-0092-017) [46]. Previously isolated TSs were also prepared and included for this study (TS13-30, TS13-64, and TS15-88). The media is composed of DMEM/F-12 (Mediatech, Manassas, VA, USA), 1× B27 (Invitrogen, San Diego, CA, USA), 20 ng/mL basic fibroblast growth factor, and 20 ng/mL epidermal growth factor (Sigma-Aldrich, St. Louis, MO, USA) [39–44, 47, 48]. Patient-derived GSC11 GBM TS were kindly provided by Frederick F. Lang’s laboratory (The University of Texas MD Anderson Cancer Center) [40, 49, 50]. Normal human astrocyte (NHA) was purchased from LONZA (Catalog number CC-2565). Culture conditions for GSC11 TSs and human astrocytes were the same as above. TS mutation profiles were extrapolated from the profiles of matching tumor tissues, and TS13-64 was profiled by RNAseq.

### DNA pyrosequencing

All GBM tissue specimens were examined by modified pyrosequencing to evaluate *MGMT* promoter methylation status in the hospital setting [51]. DNA was extracted from diagnostic formalin-fixed, paraffin-embedded (FFPE) GBM samples using a Maxwell CSC DNA FFPE Kit (Promega, USA). The annealing temperature was 53 °C, and samples were analyzed on a Pyromark Q24 MDx System (Qiagen, Germany). To categorize tumors based on *MGMT* promoter methylation status, we used a threshold of < 8% for the average percentage of four CpG sites in exon 1 [51].

### Mutation calling

Formalin-fixed, paraffin-embedded (FFPE) tissue blocks were sequenced with Trusight Tumor 170 panel (Illumina, United States) [52]. Maxwell CSC DNA FFPE Kit (Promega, United States) was used to prep for DNA/RNA hybrid capture in the Nextseq 550 Dx (Illumina). Trusight Tumor 170 App Pipeline was used to analyze DNA small variants with *Homo* *sapiens* hg19 genome as the reference (Homo sapiens, UCSC). Exonic mutations that passed Illumina QC filter were included. Mutations less than 100 depth or less than 3% of variant allele frequency were excluded from the analysis.

### Transcriptome data analysis

The samples of TSs for RNAseq were hybridized with All Human V6 + UTR baits (individual TS, *n* = 20; for TMZ treatment, triplicated TS13-64 and GSC11). All of the transcripts in this analysis were merged and labeled after same alignment and counting process. GSC11 and TS13-64 TS samples (with TMZ) were analyzed in the same manner. Gene expression level data were calculated by summing up the transcripts in the gene location (GRCh38.p5). Controversial transcripts were reconfirmed in the sequence level that is extracted from gffread (-w option) [53]. An unsupervised selection of the expressed genes (Coefficient of variation > 10) were included for the t-SNE analysis [54].

### Single cell RNAsequencing

GBM-derived TS 13–64 maintained under spheroid cell culture condition with B27. Within 30 min before the single-cell RNAsequencing (scRNAseq), the cells were dissociated with accutase. The 10× Genomics Chromium platform was used to capture and barcode the cells to generate single-cell Gel Beads-in-Emulsion (GEMs) by following the manufacturer’s protocol. scRNAseq expression data were analyzed with Seurat v2.3.4 (PCA, Cluster, t-SNE and cluster). In brief, the Seurat object was generated from digital gene expression matrices. To maintain the *TERT* positive cells, the filtering of the number of genes detected in each cell was not restricted. The percent of mitochondrial genes were not restricted in our analysis. Normalized scaled data was found to have two distinct clusters. Shared nearest neighbor (SNN) modularity optimization-based clustering algorithm revealed two to seven clusters depending on the resolution variable (from 0.01 to 0.5). Receiver operating characteristic (ROC) was used for the identification of differentially expressed genes for each class with log fold change 0.25. We examined the area under the ROC curve (myAUC) with two and three cluster models.

### Gene set enrichment analysis (GSEA)

Genes used in enrichment analysis were selected based on their coefficients of variation [the variance divided by the mean across the comparison group (*n* > 1) and mean expression (> 5 fragments per kilobase of transcript per million mapped reads (FPKM)] at the transcript level. Statistically significant genes were included in GSEA using the Reactome and KEGG database, with a significance threshold of *p* < 0.01 [55, 56]. Pathway significance was calculated as the − log10 of the analysis *p*-value.

### Validation sets

Gene expression level data of TCGA GBM was collected from Xena browser (University of California, United States) [57]. Survival data was gathered from the TCGA GBM, which was processed by GEPIA homepage [58]. Long non-coding RNA list of cancer was obtained from the Gold lab homepage [59].

### Temozolomide treatment

The TSs (TS13-64 and GSC11) were dissociated using accutase (Invitrogen, United States) to the single cells [60]. After 1 day of stabilization, TMZ 250 µM was added for 1 × 10^6^ cells/100 mm^3^ dish in triplicate. After 72 h, the plates were harvested separately for the RNAseq.

### Statistical analysis

For the group comparison in the Table 1, we used Pearson’s Chi-squared test with Yates’ continuity correction. Wilcoxon, Kruskal–Wallis, and Student’s *t*-tests were used for intergroup comparisons of gene expression levels. For the scatter plot, Pearson correlations were calculated for individual groups using the ggpubr package in R (v. 0.4.0). For cell data, *p* < 0.05 was regarded as significant (by *t*-test). In two-group comparisons, genes with *p* < 0.0001 by Student’s *t*-test were regarded as significant and included in the heatmaps.

**Table 1:** Baseline characteristics of IDH-wildtype GBM and its derived TSs

	Tumor samples (n = 58)	GBM TSs (n = 23)	p-value*
Age	58.9 ± 12.4	59.7 ± 11.0	0.78
Sex			0.66
Male	33	15	
Female	25	8	
MGMT promoter			0.67
Methylation	23	11	
Unmethylation	35	12	
TP53			0.027
Mutant	28	16	
Wildtype	30	4	
Unknown		3	
TERT promoter			0.30
Mutant	44	18	
Wildtype	14	2	
Unknown		3	
PTEN			0.80
Mutant	31	12	
Wildtype	27	8	
Unknown		3	
EGFR			0.99
Alterations	28	9	
Wildtype	30	11	
Unknown		3	

All samples are primary glioblastomas or its derived TSs. Presentation of age with mean ± standard deviation

GBM: glioblastoma, IDH: isocitrate dehydrogenase, TSs: GBM tumorspheres, MGMT: *O*-6-methylguanine-DNA methyltransferase, TP53: tumor protein p53 gene mutation, TERT: telomerase reverse transcriptase, PTEN: phosphatase and tensin homolog, EGFR: epidermal growth factor receptor

* p-value compared IDH-WT GBM tumor samples and GBM TSs (GSC11 or normal human astrocyte are excluded from this table)

### Data availability

The tumor tissue and TS datasets (Severance cohort) analyzed during the current study will be published in the Arrayexpress and GEO databases. The cancer genome atlas (TCGA) data from the gene expression profiling interactive analysis (GEPIA, v. 1) and cBioportal databases were included after data analysis to validate gene correlation [58, 61].

## Results

### TS isolation from the *IDH*-WT GBM tissues

In this bioinformatics analysis, each tumor tissue was non-selectively cultured to establish GBM TSs for RNAseq (Table 1) [44]. We found that *TP53*-mutant *IDH*-WT GBM comprises 48% of samples (28/58) which is relatively consistent with the reports [16].

Severance cohort of TSs revealed *TP53* mutation status may be associated with the isolated TS (Table 1). Most of the isolated TSs are *TP53* mutant (80%, 16/20, 3 sample excluded for the absence of next-generation sequencing data). The frequency of the *TP53* mutants is different from that of GBM tissue (*p* = 0.027, Table 1). Other variables show no difference between the tissue and TSs (Table 1). *TERT* mutant TSs were found in the 90% of samples, however its composition ratio was consistent with a literature [29].

### Gene level analysis of GBM TSs

**Fig. 1:**
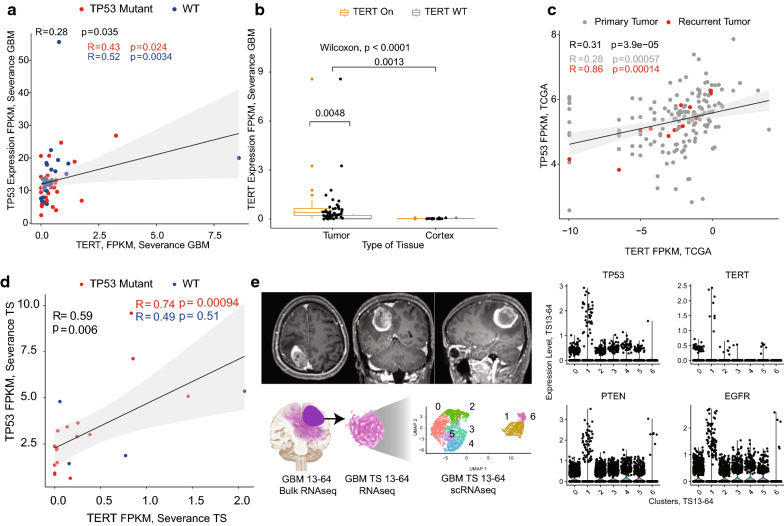
Gene expression of *TERT* and *TP53* are associated in the multiple databases. **a** Correlation of two genes in the Severance RNAseq of IDH-WT GBM tissue by *TP53* mutation status (Tumor with *TP53* mutation status, n = 58, Pearson correlation). **b** *TERT* gene expression by the mutation status of *TERT* promoter. **c** Correlation TCGA GBM in the primary and recurrent tumor. **d** *TERT* and *TP53* in the GBM tumorsphere RNAseq (TS with *TP53* mutation status, n = 20). **e** Single cell RNAseq of a representative GBM TS with multiple clusters (Modularity optimizer 1.3.0, Resolution = 0.5, Number of communities = 7). GBM TS was isolated with serum free B27 medium (see “Methods” for details)

From this finding, we examined the GBM tissue and TSs by these molecular markers: *TERT*-*TP53* correlation was found in the Severance GBM database (Fig. 1). RNAseq revealed *TERT* and *TP53* may be associated regardless of *TP53* mutation status (Fig. 1a). Even though, *TERT* is overexpressed in the GBM tumor and *TERT* promoter mutated samples (Fig. 1b), these two gene expression levels were more associated in the recurrent GBM (Fig. 1c). GBM TSs also showed stronger association, especially in the *TP53* mutant TSs (Fig. 1d). Single cell RNAseq revealed these two genes, as well as other known driver genes, are overexpressed in a single cluster (Fig. 1e).

**Fig. 2:**
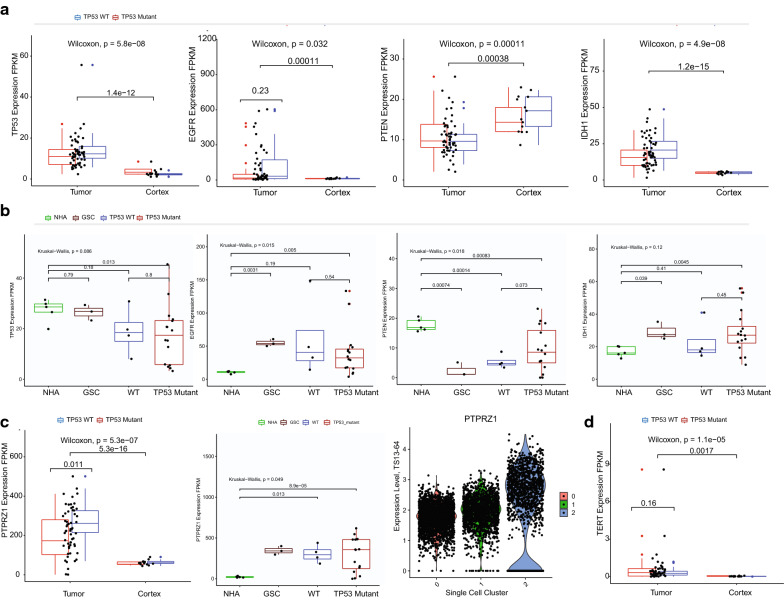
Driver-associated genes are translated from GBM tissues to TSs. We illustrated the gene expression pattern of *TP53*, *EGFR*, *PTEN*, IDH1, *PTPRZ1*, and *TERT* by the mutation status. **a** Gene expression of IDH-WT GBM and tumor-free cortical control tissues (Tumor, n = 58; Control, n = 24). **b** GBM TSs (TSs with the mutation status, n = 20) derived from the *IDH*-wildtype GBM tissues and controls (NHA, n = 3; GSC11, n = 3). **c** *PTPRZ1* is illustrated with a single cell RNAseq cluster data (Right panel, three clusters are further described in the Fig. 5). **d** *TERT* expression is grouped by the *TP53* mutation status in the tissues. White background indicates tissue bulk RNAseq. Blue background indicates the FPKM expression in the cell bulk RNAseq

**Fig. 3:**
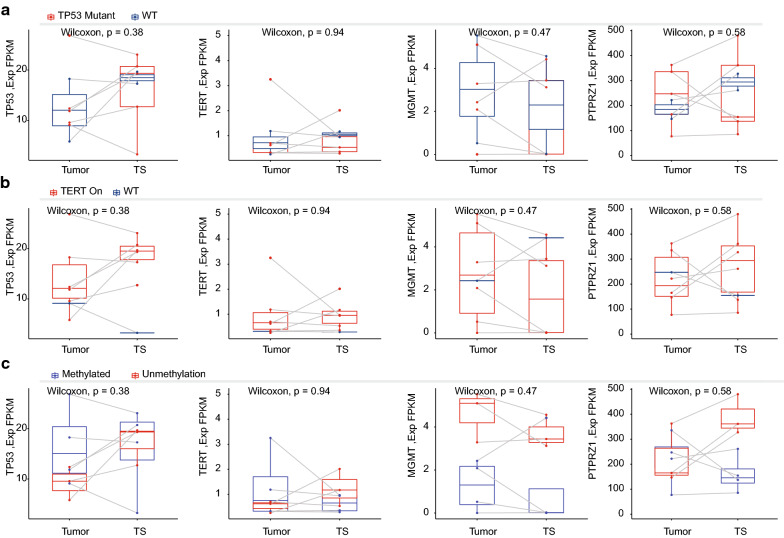
Gene expression patterns of the matched GBM TSs with the original GBM tissues. Gene expression profiles of *TP53*, *TERT*, *MGMT*, and *PTPRZ1* were displayed in the matching samples (n = 7 for each group). **a** Expression grouped by *TP53* mutation status. **b** Gene expression by *TERT* promoter mutation status. **c** Gene expression by the *MGMT* promoter methylation status

We examined the *IDH*-WT GBM tissues and GBM TSs, whether these samples are associated by other factors (Figs. 2, 3). We found *TP53*, *EGFR*, IDH1, *PTPRZ1*, and *TERT* are significantly overexpressed in the tumor tissue than the control (Fig. 2). However, these gene expression levels were not different by the *TP53* mutation status in the tissue (Fig. 2a). GBM TSs showed *EGFR*, *PTEN*, IDH1, *PTPRZ1* were overexpressed in the TSs than the normal human astrocytes (NHAs). However, *TP53* gene was not showing elevated trend than the NHAs (Fig. 2b).

Downregulated trend of *PTEN* expression in the GBM tissue than the cortex (Fig. 2a) is reflected in the GSC11 and GBM TSs (Fig. 2b). As the *BAX*, *CDKN1A*, and *MIR34AHG* was associated to be elevated in the *TP53* mutation status [21, 62, 63]: *BAX*, *CDKN1A*, and *MIR34AHG* are overexpressed in the *IDH*-WT GBM tissue than the cortex. However, there was no trend in the TSs by the *TP53* mutation status (Additional file 1).

There were seven matching samples of GBM tissues and TSs (Fig. 3). We evaluated the gene expressions of *TP53*, *TERT*, *MGMT* and *PTPRZ1* by the molecular markers: *TP53* mutation status was not associated with the conservation of the gene expression levels (Fig. 3a). *TERT* promoter mutation was associated with the higher expression of *TERT* and *TP53* gene in the tissue and TSs (Fig. 3b). *MGMT* promoter methylation status was associated with the *MGMT* gene expression (Fig. 3c).

### Transcriptomic level analysis of GBM TSs

**Fig. 4:**
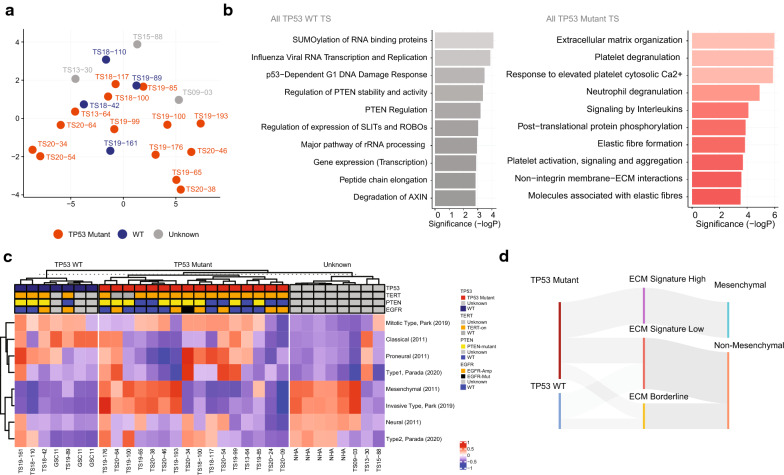
Transcriptomic signatures of GBM TSs by the *TP53* mutation status. **a** t-SNE plot of the most variable genes in the GBM TSs (An unsupervised, expression-based criteria. Details in the method). **b** Reactome gene set enrichment analysis by the *TP53* mutation status. Two TSs (TS-20–24 and 20–09) were excluded from the analysis). **c** Gene set variation analysis with the reported GBM subtype genes. **d** River plot showing the relation among the *TP53* mutation status, ECM signature, and the Verhaak subtypes

We examined whether the *TP53* mutant TSs are different from the *TP53* WT TSs (Fig. 4). Unsupervised gene variability-based t-SNE showed no significant difference by the *TP53* mutation status (Fig. 4a). There was no definite difference by other molecular markers (Additional file 2). In our TSs, *TP53* mutant TSs were relatively more heterogeneous than the *TP53* WT TSs (Fig. 4c). Combining the results of DEG and GSVA, we found the ECM-related signatures came from the mesenchymal subtype of GBM TSs (Fig. 4b–d, Additional file 3).

Additionally, we found no definite association of the gene sets of invasion (or EMT, detail in the method) and glioma neurosphere with the *TP53* mutation status of GBM TSs [64, 65]. EMT genes were not definitely associated with *TP53* mutation status (Additional file 4). Majority of the TS followed the glioma cell expression patterns: Glioma sphere downregulated genes (77%, 17/22, Additional file 5a) and upregulated genes (54%, 12/22, Additional file 5b) [65].

### Single cell level RNAseq analysis of GBM TS

**Fig. 5:**
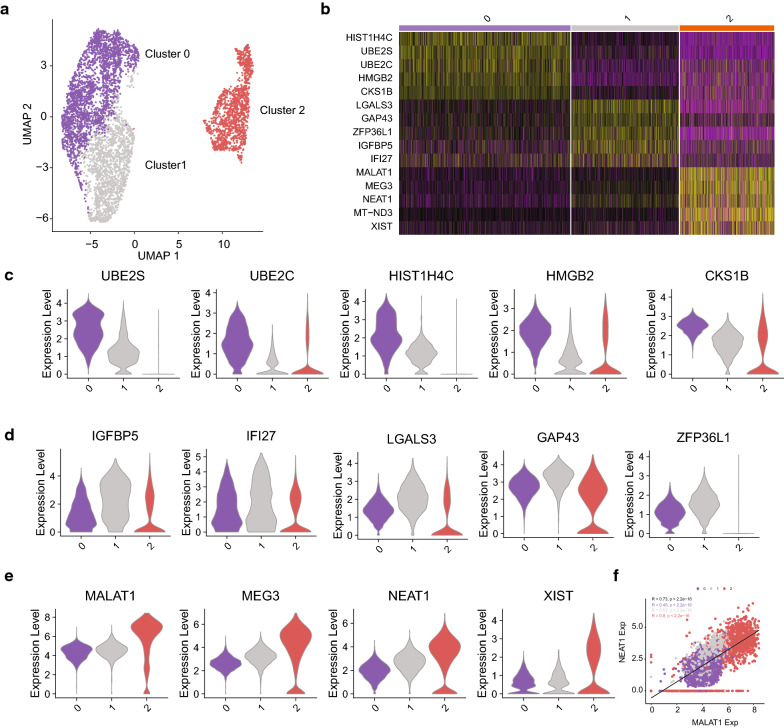
Single cell RNAsequencing of GBM TS13-64. **a** UMAP colored with the three clusters. **b** Top DEGs in each group. **c** Violin plot of the group 0 DEG. **d** Violin plot of the group 1 DEG. **e** Violin plot of the group 2 DEG. **f** Scatter plot comparing two gene expressions in the single cell level

One GBM TS (13–64) was selected for the single cell RNAsequencing (Figs. 1e, 5). The TS was derived from a 56-year-old female patient with no medical history except a carrier status of hepatitis B virus. Her chief complaint was weakness on the left arm and leg for 2 weeks. Magnetic resonance imaging (MRI) found an invasive phenotype on the MRI with high gadolinium-enhancing mass (Right parietal lesion, 4.93 cm in diameter) surrounded by extensive T2 FLAIR high density [1]. Pathology confirmed the diagnosis of GBM. The initial molecular phenotype of this tumor was IDH-wildtype, *MGMT* promoter unmethylated status, and 1p intact/19q intact. UMAP clustering showed two distinct clusters. DEGs from three cluster showed a cluster was enriched with driver genes and long noncoding RNAs (Fig. 5). Interestingly, in a non-mixed immortal TS line (TS13-64), a driver genes-enriched cluster occupied small number of cells and larger portion was not expressing the driver-associated genes (Fig. 1e, Additional file 6).

Most of the DEGs are not exclusively expressed in a single cluster (Fig. 5c–e). Single cell level correlation between *MALAT1* and *NEAT1* was found in the three clusters (Fig. 5f).

### Transcriptomic change after TMZ treatment regardless of *TP53* mutation status

**Fig. 6:**
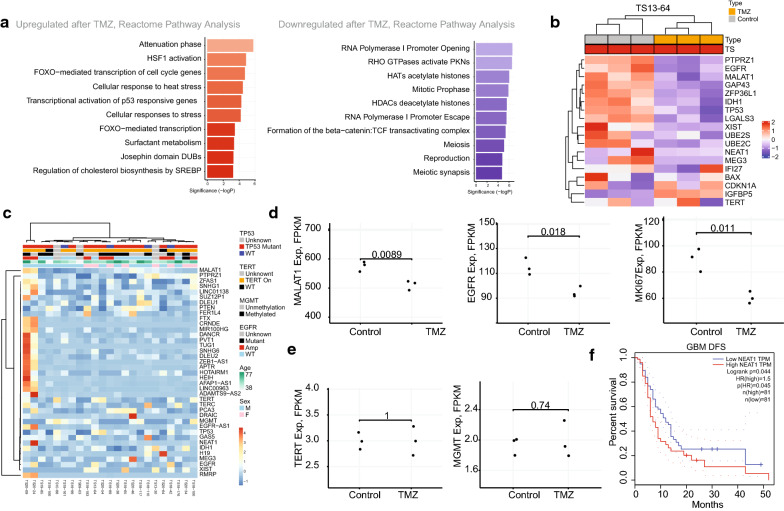
TMZ treatment downregulated most of the single cell derived DEGs. **a** Reactome analysis of the commonly altered genes in the two GBM TSs: TS13-64 and GSC11. **b** The TMZ treated TS13-64 with the single cell derived DEGs and the known driver-associated genes. **c** Overall heatmap of the single cell derived DEGs and the known driver genes in the TS. **d** Examples of the downregulated genes by the TMZ. **e** Examples of the relatively stable genes by the TMZ. **f** Disease-free survival by the *NEAT1* gene in the GBM [58]

We examined whether TMZ can change the gene expression pattern of GBM TS (13–64) and GSC11 (Fig. 6a). We included a driver gene expression matched GSC11 as control. A difference of GSC11 and TS13-64 was *TP53* mutation status (Additional file 7). Same amount of TMZ on the same number of cells showed an elevated stress-associated response with *CDKN1A* and downregulation of *KIF20A* gene expression (Fig. 6a, Additional file 8) [66].

Gene level downregulation was found in the multiple driver-associated genes and the DEGs from the single cell RNAseq of TS13-64 (Fig. 6b). TS13-64 was not distinct from other GBM TSs in the base expression level (Fig. 6c). In addition to the downregulated genes (Fig. 6d), there were relatively stable genes (Fig. 6e) and upregulated genes such as *IGFBP5* (Fig. 6). In our study, TMZ treatment not definitely changed the level of *NEAT1*. Among the genes of Fig. 6b, *NEAT1* was found to be associated with progression-free survival (PFS) in the GBM database (Fig. 6f). Furthermore, the median level of *NEAT1 *was associated with poor overall survival and poor PFS in the lower grade gliomas (Additional file 9). *NEAT1* expression was elevated in the *TP53* wildtype GBM tissues of Severance cohort, but other molecular markers were not associated with the gene level (Additional file 10).

## Discussion

*TP53* mutation is one of the most common alterations across tumor types [22], and is observed in the early stages of GBM, along with changes in related pathways [17, 67, 68]. However, its association with GBM TS isolation rate was not reported yet [1, 41, 44, 47, 69–71]. In this retrospective analysis, we found that *TP53* mutants were more amenable to isolation from tissue (Table 1). RNAseq data shows that *TP53* mutants are overexpressing ECM related genes with more mesenchymal subtypes (Fig. 4). CCLE database shows, no tendency by the *TP53* mutation status suggesting this finding may be examined in the prospective study (Additional file 11). The details of the DEGs for Fig. 4 are included in Additional file 12.

Using *IDH*-WT GBM-derived TSs, we found a positive association between the levels of *TERT* and *TP53*. Furthermore, GBM tissues also displayed this association (Fig. 1) [58, 61], and other publicly available data suggest that *TERT* and *TP53* are associated in other tumor types and normal brain tissue, such as lower grade glioma, head and neck cancer, and acute myeloid leukemia [58]. However, not all cancer types exhibit this association (for example, urothelial bladder carcinoma) [58]. In retrospective analysis, our TSs are biased to the *TP53* mutants, and the correlation of *TERT* and *TP53* may be examined in the balanced dataset (Fig. 1d). In an attempt to examine the response of TSs to TMZ, we planned a comparison of two cells which are only different by the *TP53* mutation status (TS13-64, *TP53 *mutant; GSC11, *TP53 *wildtype; Additional file 13). The expression pattern and response to TMZ of *IGFBP5, *which is commonly upregulated in GBM than cortical tissues, may need attention as it has been studied as one of important factors of GBM and gliomas (Fig. 6b). About the association between *TP53 *and *NEAT1 *(Additional file 10), we need more evidences whether they are correlated in biological manner.  

*TP53* mutations are known to have gain-of-function effects in GBM cells [18], and the abundance of *TP53* mutant TSs in this TS RNAseq data may be associated to  a survival benefit to the TSs. However, we emphasize the overrepresentation of *TP53* mutant TSs than the WT TSs does not provide a direct evidence of the gain-of-function effect of the mutation.

Even with these limitations, our study indicates a clue to approach the in vitro models of glioma with the expression pattern of driver genes (including the MGMT promoter methylation status, Additional file 14): not all cells in a glioma TS are directly associated with the driver-associated genes (Fig. 5a). The culture of glioblastoma sphere cell (GSC) became a well-established laboratory technique [72–74]. For example, GSC11 was established from the fresh surgically operated GBM tissue, and are used for drug screening or transcriptome analysis [49, 75, 76]. These cells become necrotic in the orthotopic models and organoid models [77, 78]. Established TSs was believed to have stemness, the potential to form orthotopic tumors, and their characteristics do not change with repeated subculture [39–43, 45, 47, 69]. These spheroid cultures of tumor cells however, was not a single homogeneous group of cells (Fig. 5a) [42, 45, 79]. Furthermore, the result of GSEA should not be regarded as the best representation of a transcriptomic status (Additional file 15 of KEGG database shows different pattern with the same list of genes from Fig. 4b). 

Finally, we rediscovered *NEAT1* and other long noncoding RNAs (LncRNAs) that are important in the cancer biology as well as in GBM cell growth and invasion [59, 80]. Furthermore, *NEAT1* distinguishes the survival both in the GBM and lower grade gliomas (Additional file 9). Our data also shows *NEAT1* is overexpressed in the driver-enriched cluster of a GBM TS (Fig. 4). We examined the value of myAUC in the three cluster model of Fig. 5a (Additional file 16): The cluster 2, which was enriched with other driver genes, harbors five cancer related LncRNAs as the characterizing genes. Using the driver genes as the marker, we claimed the TSs are reflecting the characteristics of GBM tissue, at least with the driver gene expression. From bulk TSs to single individual cell level of a TS, we found these driver genes are expressed in a single cluster which has LncRNA classifiers (Additional file 16). When *NEAT1* and accompanying LncRNAs are searched in the public datasets, however, the glioblastoma tissue is not seem to be enriched with these genes. Our new finding with scRNAseq and LncRNAs may help neglected LncRNAs to be included for a research theme.

## Conclusion

We found that GBM TSs represent the tissue level gene expression patterns of *EGFR*, *TERT*, *PTEN*, *IDH1*, *PTPRZ1,* and *MGMT*. Single cell sequencing revealed these driver-associated genes are co-expressed with the cancer driver noncoding genes. Our data shows the association of the protein coding driver genes and the non-coding driver genes.

## Supplementary information

**Additional** **file** **1.** Gene expression profiles of CDKN1A, BAX, and MIR34AHG (Related to the Fig. 3). Three genes are overexpressed in the GBM tumors than the control tissues. However, there was no definite difference by the *TP53* mutation status in the tissues and the TSs. a. CDKN1A and BAX. b. MIR34AHG.

**Additional** **file** **2.** t-SNE of the GBM TSs (Related to the Fig. 4). a. t-SNE plot with additional samples than Fig. 4a. (Upper) Two TSs (TS20-24 and 20-09) are added to Fig. 4a. (Lower) In addition to the upper panel, NHAs and GSC11 are added. (b–f). t-SNE plot for comparison by the molecular markers. b. *TP53* mutation status. c. *TERT* promoter mutation status. d. *MGMT* promoter methylation status. e. *PTEN* mutation status. f. *EGFR* alteration status.

**Additional** **file** **3.** Gene expression heatmap of the extracellular matrix related gene set (Related to the Fig. 4). This gene set was obtained from a *TP53* mutant TS-related Reactome analysis of Fig. 4b.

**Additional** **file** **4.** mSig DB genes of epithelial mesenchymal transition (Related to the Fig. 4). The criteria of selecting these genes are described in the additional method section.

**Additional** **file** **5.** TS gene expression heatmap of the glioma sphere gene sets (Related to the Fig. 4). a. Glioma sphere downregulated genes. b. Glioma sphere upregulated genes [65]. TS: Tumorsphere.

**Additional** **file** **6.** Violin plots of gene expression (Related to the Fig. 5). a. NOTCH pathway related genes. b. Neurotransmitter related genes. c. Glioma type related genes.

**Additional** **file** **7.** Detailed description of the methods.

**Additional** **file** **8.** TS13-64 and GSC11 are treated with TMZ (Related to the Fig. 6). a. Based on the driver-associated gene expressions, we selected two GBM TSs. b. Two types of TSs are sent for RNAseq. c. Both cells are showing elevated CDKN1A and downregulated *KIF20A* after TMZ (Gene set enrichment assay of these two cells are displayed in Fig. 6a).

**Additional** **file** **9.** Survival plots of GBM by *NEAT1* (Related to the Fig. 6f). a. Overall survival by the median expression of *NEAT1* in the TCGA GBM (processed in GEPIA). b. The results of the lower grade glioma database [58].

**Additional** **file** **10.** Gene expression of *NEAT1* in the Severance database (Related to the Fig. 6). The gene expression of *NEAT1* was compared by the molecular markers in the IDH-WT GBM RNAseq data.

**Additional** **file** **11.** Subtypes of the CNS related tumor cells in the CCLE database (Related to the Fig. 4). The RNAseq data of CCLE was downloaded and analyzed by the same method for the subtype analysis (Related to Fig. 4c). Both group of *TP53* mutation status cells were classified to mesenchymal (or invasive) types [1, 81].

**Additional** **file** **12.** The list of differentially expressed genes by *TP53* mutation status. Data calculated by the Reactome database.

**Additional** **file** **13.** GBM oncogene mutation profiles. The mutation profiles of the two GBM TSs (TS13-64, GSC11).

**Additional** **file** **14.** *MGMT* gene expression from the tissue, GBM TSs, and TS13-64 (Related to the Fig. 3). a. Gene expression by *MGMT* promoter methylation status in the GBM (n = 58; Unmethylated samples, n = 35; Methylated Samples, n = 23) and its associated control cortex tissue (n=24). GBM TSs is displayed in the right panel (Unmethylated TS n = 12, Methylated TS n = 11).

**Additional** **file** **15.** KEGG analysis on the GBM TSs by the mutation status of *TP53* (Related to the Fig. 4). Each gene of GBM TSs (not excluding TS20-24 and 20-09) were calculated for the gene set enrichment analysis. The highly enriched gene lists were examined with the KEGG database.

**Additional** **file** **16.** AUC values from the single cell RNAseq (Related to the Figs. 5 and 6). The differentially expressed genes from the clusters of TS13-64 were obtained from Seurat algorithm. Regardless of the resolution parameters (or the number of clusters), the driver-gene enriched cluster was always marked with the glioma related long noncoding RNAs [59].

## Data Availability

The tumor tissue and TS datasets (Severance cohort) analyzed during the current study will be published in the Arrayexpress and GEO. TCGA data of GEPIA and cBioportal was included after analysis of data for the validation of gene correlation [58, 61].

## Abbreviations

B27: Neurobasal media
DEG: Differential gene expression
*EGFR*: Epidermal growth factor receptor
EMT: Epithelial–mesenchymal transition
Exp: Row-wise group mean expression of raw FPKM of RNAseq
FDR: False discovery rate
FPKM: Fragments Per Kilobase Million
GBM: Glioblastoma
*IDH*: Isocitrate dehydrogenase
IHC: Immunohistochemistry
*MGMT*: *O*-6-Methylguanine-DNA methyltransferase
*MGMT*p: *MGMT* Promoter methylation status
*PTEN*: Phosphatase and tensin homolog
scRNAseq: Single cell RNA sequencing
TCGA: The cancer genome atlas
*TERT*: Telomerase reverse transcriptase
*TERT* On: The promoter of telomerase reverse transcriptase (*TERT*) is mutated in the tumor sample with targeted sequencing (*TERT* Off indicates the WT of *TERT* promoter regions)
*TP53*: Tumor protein p53 gene mutation
TS: Tumorsphere
TSs: Tumorspheres
TMZ: Temozolomide
WT: Wildtype

## References

[1] Park J, Shim J-K, Yoon S-J, Kim SH, Chang JH, Kang S-G (2019). Transcriptome profiling-based identification of prognostic subtypes and multi-omics signatures of glioblastoma. Sci Rep.

[2] Louis DN, Perry A, Reifenberger G, von Deimling A, Figarella-Branger D, Cavenee WK (2016). The 2016 World Health Organization Classification of Tumors of the Central Nervous System: a summary. Acta Neuropathol.

[3] Puchalski RB, Shah N, Miller J, Dalley R, Nomura SR, Yoon JG (2018). An anatomic transcriptional atlas of human glioblastoma. Science.

[4] Tirosh I, Suvà ML (2020). Tackling the many facets of glioblastoma heterogeneity. Cell Stem Cell.

[5] Verhaak RGW, Hoadley KA, Purdom E, Wang V, Qi Y, Wilkerson MD (2010). Integrated genomic analysis identifies clinically relevant subtypes of glioblastoma characterized by abnormalities in PDGFRA, IDH1, EGFR and NF1. Cancer Cell.

[6] Patel AP, Tirosh I, Trombetta JJ, Shalek AK, Gillespie SM, Wakimoto H (2014). Single-cell RNA-seq highlights intratumoral heterogeneity in primary glioblastoma. Science.

[7] Brat DJ, Aldape K, Colman H, Holland EC, Louis DN, Jenkins RB (2018). cIMPACT-NOW update 3: recommended diagnostic criteria for "Diffuse astrocytic glioma, IDH-wildtype, with molecular features of glioblastoma, WHO grade IV". Acta Neuropathol.

[8] Yan H, Parsons DW, Jin G, McLendon R, Rasheed BA, Yuan W (2009). IDH1 and IDH2 mutations in gliomas. N Engl J Med.

[9] Ceccarelli M, Barthel FP, Malta TM, Sabedot TS, Salama SR, Murray BA (2016). Molecular profiling reveals biologically discrete subsets and pathways of progression in diffuse glioma. Cell.

[10] Jung E, Alfonso J, Monyer H, Wick W, Winkler F. Neuronal signatures in cancer. Int J Cancer. 2020;n/a(n/a). 10.1002/ijc.33138 32510582

[11] Roh TH, Park HH, Kang SG, Moon JH, Kim EH, Hong CK (2017). Long-term outcomes of concomitant chemoradiotherapy with temozolomide for newly diagnosed glioblastoma patients: a single-center analysis. Medicine.

[12] Nobusawa S, Watanabe T, Kleihues P, Ohgaki H (2009). IDH1 Mutations as molecular signature and predictive factor of secondary glioblastomas. Clin Cancer Res.

[13] Luchman HA, Stechishin OD, Dang NH, Blough MD, Chesnelong C, Kelly JJ (2012). An in vivo patient-derived model of endogenous IDH1-mutant glioma. Neuro Oncol.

[14] Rohle D, Popovici-Muller J, Palaskas N, Turcan S, Grommes C, Campos C (2013). An inhibitor of mutant IDH1 delays growth and promotes differentiation of glioma cells. Science.

[15] Piaskowski S, Bienkowski M, Stoczynska-Fidelus E, Stawski R, Sieruta M, Szybka M (2011). Glioma cells showing IDH1 mutation cannot be propagated in standard cell culture conditions. Br J Cancer.

[16] Olivier M, Hollstein M, Hainaut P (2010). TP53 mutations in human cancers: origins, consequences, and clinical use. Cold Spring Harb Perspect Biol.

[17] Lee JH, Lee JE, Kahng JY, Kim SH, Park JS, Yoon SJ (2018). Human glioblastoma arises from subventricular zone cells with low-level driver mutations. Nature.

[18] Olafson LR, Gunawardena M, Nixdorf S, McDonald KL, Rapkins RW (2020). The role of TP53 gain-of-function mutation in multifocal glioblastoma. J Neurooncol.

[19] Amit M, Takahashi H, Dragomir MP, Lindemann A, Gleber-Netto FO, Pickering CR (2020). Loss of p53 drives neuron reprogramming in head and neck cancer. Nature.

[20] Amundson SA, Do KT, Vinikoor LC, Lee RA, Koch-Paiz CA, Ahn J (2008). Integrating global gene expression and radiation survival parameters across the 60 cell lines of the National Cancer Institute Anticancer Drug Screen. Cancer Res.

[21] Amundson SA, Myers TG, Scudiero D, Kitada S, Reed JC, Fornace AJ (2000). An informatics approach identifying markers of chemosensitivity in human cancer cell lines. Cancer Res.

[22] Donehower LA, Soussi T, Korkut A, Liu Y, Schultz A, Cardenas M (2019). Integrated analysis of TP53 gene and pathway alterations in The Cancer Genome Atlas. Cell Rep.

[23] Pedrote MM, Motta MF, Ferretti GDS, Norberto DR, Spohr TCLS, Lima FRS (2020). Oncogenic gain of function in glioblastoma is linked to mutant p53 amyloid oligomers. iScience.

[24] Ham SW, Jeon H-Y, Jin X, Kim E-J, Kim J-K, Shin YJ (2019). TP53 gain-of-function mutation promotes inflammation in glioblastoma. Cell Death Differ.

[25] Robles AI, Harris CC (2010). Clinical outcomes and correlates of TP53 mutations and cancer. Cold Spring Harb Perspect Biol.

[26] Cho SY, Park C, Na D, Han JY, Lee J, Park OK (2017). High prevalence of TP53 mutations is associated with poor survival and an EMT signature in gliosarcoma patients. Exp Mol Med.

[27] Yuan X, Larsson C, Xu D (2019). Mechanisms underlying the activation of TERT transcription and telomerase activity in human cancer: old actors and new players. Oncogene.

[28] Nonoguchi N, Ohta T, Oh JE, Kim YH, Kleihues P, Ohgaki H (2013). TERT promoter mutations in primary and secondary glioblastomas. Acta Neuropathol.

[29] Johanns TM, Fu Y, Kobayashi DK, Mei Y, Dunn IF, Mao DD (2016). High incidence of TERT mutation in brain tumor cell lines. Brain Tumor Pathol.

[30] Bhaduri A, Di Lullo E, Jung D, Müller S, Crouch EE, Espinosa CS (2020). Outer radial glia-like cancer stem cells contribute to heterogeneity of glioblastoma. Cell Stem Cell.

[31] Qin EY, Cooper DD, Abbott KL, Lennon J, Nagaraja S, Mackay A (2017). Neural precursor-derived pleiotrophin mediates subventricular zone invasion by glioma. Cell.

[32] Venkatesh HS, Morishita W, Geraghty AC, Silverbush D, Gillespie SM, Arzt M (2019). Electrical and synaptic integration of glioma into neural circuits. Nature.

[33] Venkataramani V, Tanev DI, Strahle C, Studier-Fischer A, Fankhauser L, Kessler T (2019). Glutamatergic synaptic input to glioma cells drives brain tumour progression. Nature.

[34] Pieper RO, Costello JF, Kroes RA, Futscher BW, Marathi U, Erickson LC (1991). Direct correlation between methylation status and expression of the human O-6-methylguanine DNA methyltransferase gene. Cancer Commun.

[35] Esteller M, Garcia-Foncillas J, Andion E, Goodman SN, Hidalgo OF, Vanaclocha V (2000). Inactivation of the DNA-repair gene MGMT and the clinical response of gliomas to alkylating agents. N Engl J Med.

[36] Lee SY (2016). Temozolomide resistance in glioblastoma multiforme. Genes Dis.

[37] Hegi ME, Diserens AC, Gorlia T, Hamou MF, de Tribolet N, Weller M (2005). MGMT gene silencing and benefit from temozolomide in glioblastoma. N Engl J Med.

[38] Yi G-Z, Huang G, Guo M, Zhang X, Wang H, Deng S (2019). Acquired temozolomide resistance in MGMT-deficient glioblastoma cells is associated with regulation of DNA repair by DHC2. Brain.

[39] Jeong H, Park J, Shim JK, Lee JE, Kim NH, Kim HS (2019). Combined treatment with 2'-hydroxycinnamaldehyde and temozolomide suppresses glioblastoma tumorspheres by decreasing stemness and invasiveness. J Neurooncol.

[40] Choi J, Lee JH, Koh I, Shim JK, Park J, Jeon JY (2016). Inhibiting stemness and invasive properties of glioblastoma tumorsphere by combined treatment with temozolomide and a newly designed biguanide (HL156A). Oncotarget.

[41] Kim EH, Lee JH, Oh Y, Koh I, Shim JK, Park J (2017). Inhibition of glioblastoma tumorspheres by combined treatment with 2-deoxyglucose and metformin. Neuro Oncol.

[42] Kang SG, Cheong JH, Huh YM, Kim EH, Kim SH, Chang JH (2015). Potential use of glioblastoma tumorsphere: clinical credentialing. Arch Pharm Res.

[43] Park J, Shim J-K, Kang JH, Choi J, Chang JH, Kim S-Y (2018). Regulation of bioenergetics through dual inhibition of aldehyde dehydrogenase and mitochondrial complex I suppresses glioblastoma tumorspheres. Neuro Oncol.

[44] Sung KS, Shim J-K, Lee J-H, Kim SH, Park S, Roh T-H (2016). Success of tumorsphere isolation from WHO grade IV gliomas does not correlate with the weight of fresh tumor specimens: an immunohistochemical characterization of tumorsphere differentiation. Cancer Cell Int.

[45] Gudbergsson JM, Kostrikov S, Johnsen KB, Fliedner FP, Stolberg CB, Humle N (2019). A tumorsphere model of glioblastoma multiforme with intratumoral heterogeneity for quantitative analysis of cellular migration and drug response. Exp Cell Res.

[46] Kong BH, Park NR, Shim JK, Kim BK, Shin HJ, Lee JH (2013). Isolation of glioma cancer stem cells in relation to histological grades in glioma specimens. Childs Nerv Syst.

[47] Kwak J, Shim JK, Kim DS, Lee JH, Choi J, Park J (2016). Isolation and characterization of tumorspheres from a recurrent pineoblastoma patient: feasibility of a patient-derived xenograft. Int J Oncol.

[48] Park J, Oh SJ, Shim J-K, Roh T-H, Ji YB, Sung KS (2016). EXTH-28. 5-AMINOLEVULINIC ACID-BASED PHOTODYNAMIC THERAPY OF GLIOBLASTOMA TUMORSPHERE AND ACQUIRED RESISTANCE BY TUMOR MESENCHYMAL STEM-LIKE CELLS. Neuro Oncol.

[49] He H, Nilsson CL, Emmett MR, Marshall AG, Kroes RA, Moskal JR (2010). Glycomic and transcriptomic response of GSC11 glioblastoma stem cells to STAT3 phosphorylation inhibition and serum-induced differentiation. J Proteome Res.

[50] Kamal MM, Sathyan P, Singh SK, Zinn PO, Marisetty AL, Liang S (2012). REST regulates oncogenic properties of glioblastoma stem cells. Stem Cells.

[51] Kim YS, Kim SH, Cho J, Kim JW, Chang JH, Kim DS (2012). MGMT gene promoter methylation as a potent prognostic factor in glioblastoma treated with temozolomide-based chemoradiotherapy: a single-institution study. Int J Radiat Oncol* Biol* Phys.

[52] Na K, Kim HS, Shim HS, Chang JH, Kang SG, Kim SH (2019). Targeted next-generation sequencing panel (TruSight Tumor 170) in diffuse glioma: a single institutional experience of 135 cases. J Neurooncol.

[53] Pertea G, Pertea M (2020). GFF utilities: GffRead and GffCompare. F1000Research.

[54] van der Maaten L, Hinton G (2008). Visualizing data using t-SNE. J Mach Learn Res.

[55] Croft D, O'Kelly G, Wu G, Haw R, Gillespie M, Matthews L (2011). Reactome: a database of reactions, pathways and biological processes. Nucleic Acids Res.

[56] Kanehisa M, Furumichi M, Tanabe M, Sato Y, Morishima K (2017). KEGG: new perspectives on genomes, pathways, diseases and drugs. Nucleic Acids Res.

[57] Goldman MJ, Craft B, Hastie M, Repečka K, McDade F, Kamath A (2020). Visualizing and interpreting cancer genomics data via the Xena platform. Nat Biotechnol.

[58] Tang Z, Li C, Kang B, Gao G, Li C, Zhang Z (2017). GEPIA: a web server for cancer and normal gene expression profiling and interactive analyses. Nucleic Acids Res.

[59] Rheinbay E, Nielsen MM, Abascal F, Wala JA, Shapira O, Tiao G (2020). Analyses of non-coding somatic drivers in 2,658 cancer whole genomes. Nature.

[60] Kim HY, Lee BI, Jeon JH, Kim DK, Kang S-G, Shim J-K (2019). Gossypol suppresses growth of temozolomide-resistant glioblastoma tumor spheres. Biomolecules.

[61] Cerami E, Gao J, Dogrusoz U, Gross BE, Sumer SO, Aksoy BA (2012). The cBio cancer genomics portal: an open platform for exploring multidimensional cancer genomics data. Cancer Discov.

[62] Hu WL, Jin L, Xu A, Wang YF, Thorne RF, Zhang XD (2018). GUARDIN is a p53-responsive long non-coding RNA that is essential for genomic stability. Nat Cell Biol.

[63] Raver-Shapira N, Marciano E, Meiri E, Spector Y, Rosenfeld N, Moskovits N (2007). Transcriptional activation of miR-34a contributes to p53-mediated apoptosis. Mol Cell.

[64] Subramanian A, Tamayo P, Mootha VK, Mukherjee S, Ebert BL, Gillette MA (2005). Gene set enrichment analysis: a knowledge-based approach for interpreting genome-wide expression profiles. Proc Natl Acad Sci USA.

[65] Günther HS, Schmidt NO, Phillips HS, Kemming D, Kharbanda S, Soriano R (2008). Glioblastoma-derived stem cell-enriched cultures form distinct subgroups according to molecular and phenotypic criteria. Oncogene.

[66] Sakai R, Morikawa Y, Kondo C, Oka H, Miyajima H, Kubo K (2014). Combinatorial measurement of CDKN1A/p21 and KIF20A expression for discrimination of DNA damage-induced clastogenicity. Int J Mol Sci.

[67] Korber V, Yang J, Barah P, Wu Y, Stichel D, Gu Z (2019). Evolutionary trajectories of IDH(WT) glioblastomas reveal a common path of early tumorigenesis instigated years ahead of initial diagnosis. Cancer Cell.

[68] Yoon S-J, Park J, Jang D-S, Kim HJ, Lee JH, Jo E (2020). Glioblastoma cellular origin and the firework pattern of cancer genesis from the subventricular zone. J Korean Neurosurg Soc.

[69] Lim EJ, Kim S, Oh Y, Suh Y, Kaushik N, Lee JH (2020). Crosstalk between GBM cells and mesenchymal stem-like cells promotes the invasiveness of GBM through the C5a/p38/ZEB1 axis. Neuro Oncol.

[70] Kim H, Kim J, Yu S, Lee Y-Y, Park J, Choi RJ (2020). A Mechanism for microRNA arm switching regulated by uridylation. Mol Cell.

[71] Oh S, Yeom J, Cho HJ, Kim J-H, Yoon S-J, Kim H (2020). Integrated pharmaco-proteogenomics defines two subgroups in isocitrate dehydrogenase wild-type glioblastoma with prognostic and therapeutic opportunities. Nat Commun.

[72] Beier D, Hau P, Proescholdt M, Lohmeier A, Wischhusen J, Oefner PJ (2007). CD133(+) and CD133(−) glioblastoma-derived cancer stem cells show differential growth characteristics and molecular profiles. Cancer Res.

[73] Uchida N, Buck DW, He D, Reitsma MJ, Masek M, Phan TV (2000). Direct isolation of human central nervous system stem cells. Proc Natl Acad Sci USA.

[74] Wang Q, Hu B, Hu X, Kim H, Squatrito M, Scarpace L (2017). Tumor evolution of glioma-intrinsic gene expression subtypes associates with immunological changes in the microenvironment. Cancer Cell.

[75] Lichti CF, Liu H, Shavkunov AS, Mostovenko E, Sulman EP, Ezhilarasan R (2014). Integrated chromosome 19 transcriptomic and proteomic data sets derived from glioma cancer stem-cell lines. J Proteome Res.

[76] Yuan S, Wang F, Chen G, Zhang H, Feng L, Wang L (2013). Effective elimination of cancer stem cells by a novel drug combination strategy. Stem Cells.

[77] Yi H-G, Jeong YH, Kim Y, Choi Y-J, Moon HE, Park SH (2019). A bioprinted human-glioblastoma-on-a-chip for the identification of patient-specific responses to chemoradiotherapy. Nat Biomed Eng.

[78] Garcia C, Dubois LG, Xavier AL, Geraldo LH, da Fonseca ACC, Correia AH (2014). The orthotopic xenotransplant of human glioblastoma successfully recapitulates glioblastoma-microenvironment interactions in a non-immunosuppressed mouse model. BMC Cancer.

[79] D'Alessandris QG, Biffoni M, Martini M, Runci D, Buccarelli M, Cenci T (2017). The clinical value of patient-derived glioblastoma tumorspheres in predicting treatment response. Neuro Oncol.

[80] Chen Q, Cai J, Wang Q, Wang Y, Liu M, Yang J (2018). Long noncoding RNA NEAT1, regulated by the EGFR pathway, contributes to glioblastoma progression through the WNT/β-catenin pathway by scaffolding EZH2. Clin Cancer Res.

[81] Verhaak RGW, Hoadley KA, Purdom E, Wang V, Qi Y, Wilkerson MD (2010). Integrated genomic analysis identifies clinically relevant subtypes of glioblastoma characterized by abnormalities in PDGFRA, IDH1, EGFR, and NF1. Cancer Cell.

