# Supplementary figures and images for: Co-expression of cancer driver genes: IDH-wildtype glioblastoma-derived tumorspheres
Source: J Transl Med. 2020 Dec 14;18:482. doi: 10.1186/s12967-020-02647-8 (PMC7734785; doi:10.1186/s12967-020-02647-8)

**a**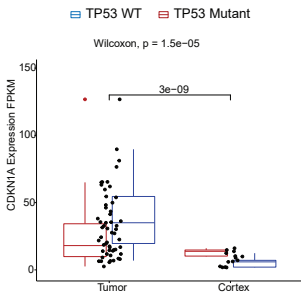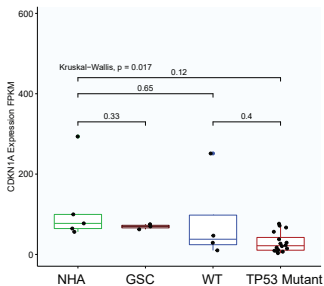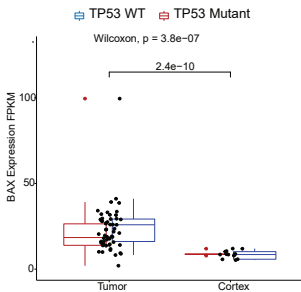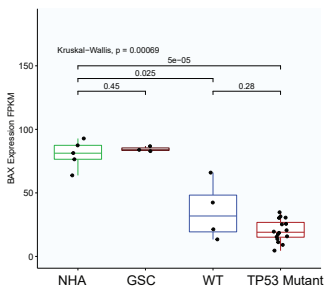**b**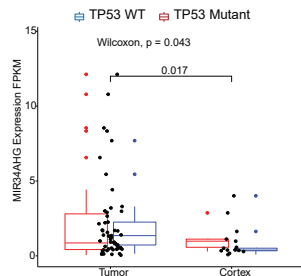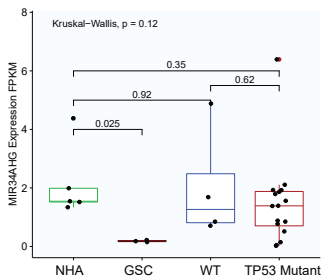

Supplement: Supplementary file 1 — Additional file 1. Gene expression profiles of CDKN1A, BAX, and MIR34AHG (Related to the Fig. 3). Three genes are overexpressed in the GBM tumors than the control tissues. However, there was no definite difference by the TP53 mutation status in the tissues and the TSs. a. CDKN1A and BAX. b. MIR34AHG. [file 12967_2020_2647_MOESM1_ESM.pdf]

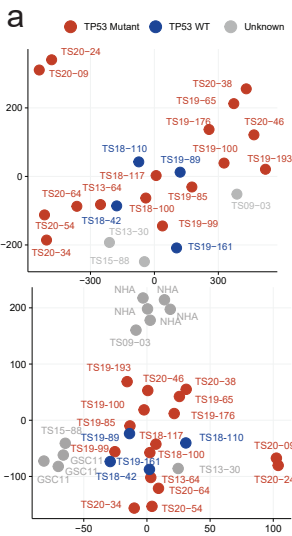

Supplement: Supplementary file 2 — Additional file 2. t-SNE of the GBM TSs (Related to the Fig. 4). a. t-SNE plot with additional samples than Fig. 4a. (Upper) Two TSs (TS20-24 and 20-09) are added to Fig. 4a. (Lower) In addition to the upper panel, NHAs and GSC11 are added. (b–f). t-SNE plot for comparison by the molecular markers. b. TP53 mutation status. c. TERT promoter mutation status. d. MGMT promoter methylation status. e. PTEN mutation status. f. EGFR alteration status. [file 12967_2020_2647_MOESM2_ESM.pdf]

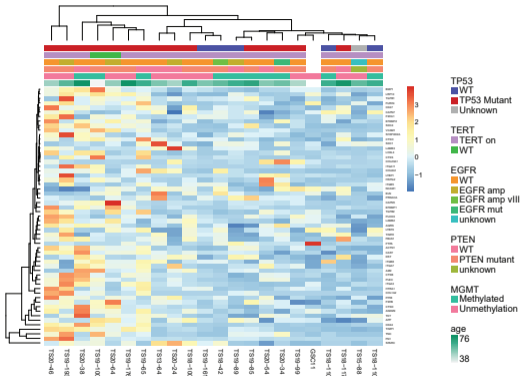

Supplement: Supplementary file 3 — Additional file 3. Gene expression heatmap of the extracellular matrix related gene set (Related to the Fig. 4). This gene set was obtained from a TP53 mutant TS-related Reactome analysis of Fig. 4b. [file 12967_2020_2647_MOESM3_ESM.pdf]

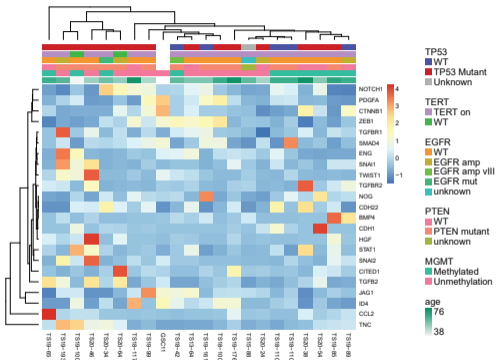

Supplement: Supplementary file 4 — Additional file 4. mSig DB genes of epithelial mesenchymal transition (Related to the Fig. 4). The criteria of selecting these genes are described in the additional method section. [file 12967_2020_2647_MOESM4_ESM.pdf]

a

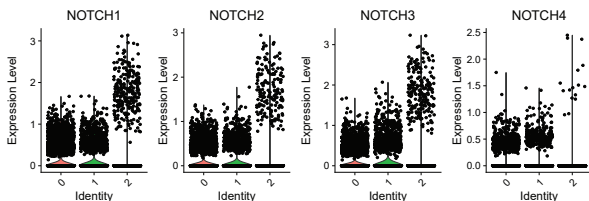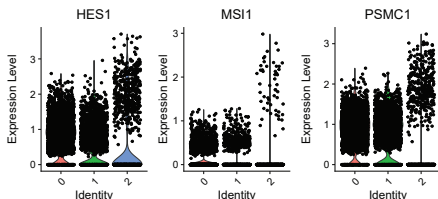

b

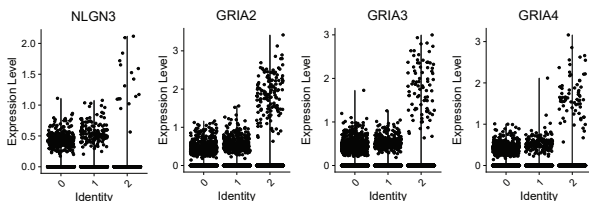

c

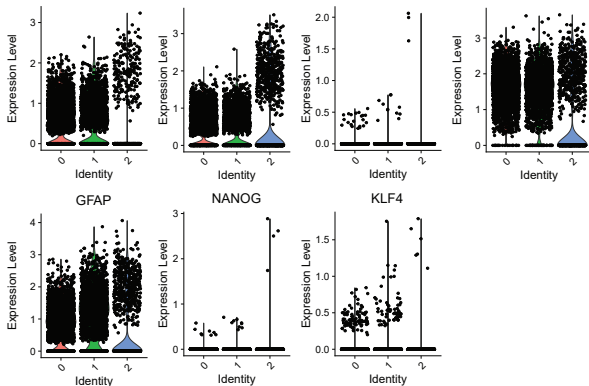

Supplement: Supplementary file 6 — Additional file 6. Violin plots of gene expression (Related to the Fig. 5). a. NOTCH pathway related genes. b. Neurotransmitter related genes. c. Glioma type related genes. [file 12967_2020_2647_MOESM6_ESM.pdf]

a

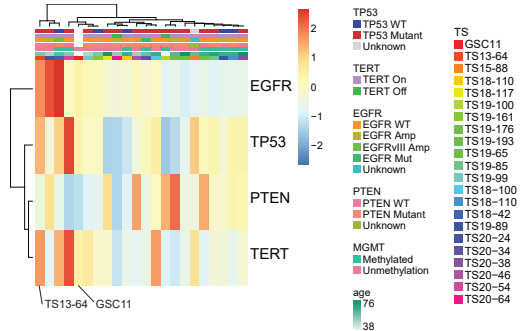

b

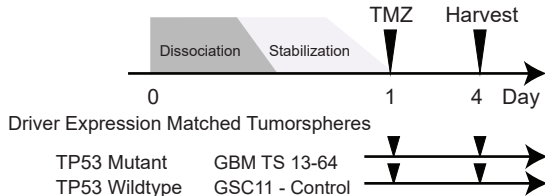

c

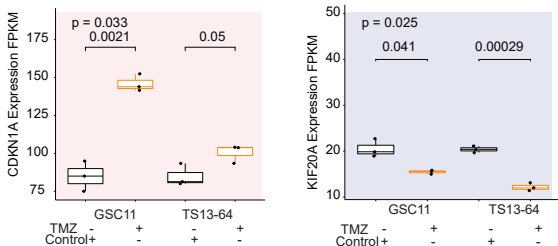

Supplement: Supplementary file 8 — Additional file 8. TS13-64 and GSC11 are treated with TMZ (Related to the Fig. 6). a. Based on the driver-associated gene expressions, we selected two GBM TSs. b. Two types of TSs are sent for RNAseq. c. Both cells are showing elevated CDKN1A and downregulated KIF20A after TMZ (Gene set enrichment assay of these two cells are displayed in Fig. 6a). [file 12967_2020_2647_MOESM8_ESM.pdf]

**a****GBM OS**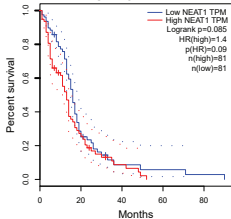**b****LGG DFS**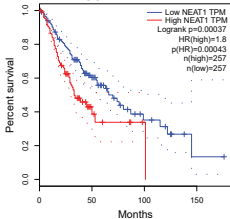**LGG OS**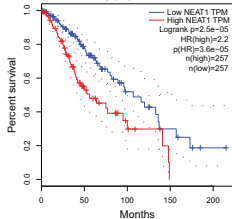

Supplement: Supplementary file 9 — Additional file 9. Survival plots of GBM by NEAT1 (Related to the Fig. 6f). a. Overall survival by the median expression of NEAT1 in the TCGA GBM (processed in GEPIA). b. The results of the lower grade glioma database [58]. [file 12967_2020_2647_MOESM9_ESM.pdf]

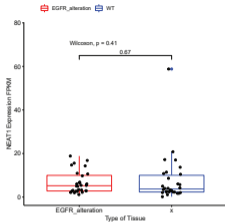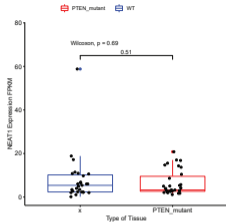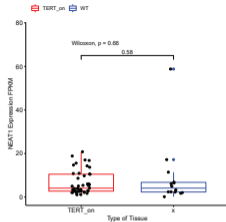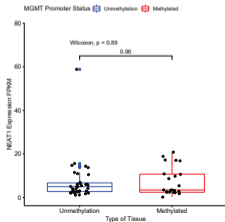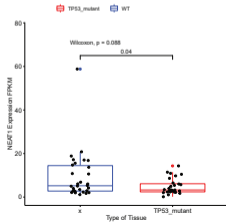

Supplement: Supplementary file 10 — Additional file 10. Gene expression of NEAT1 in the Severance database (Related to the Fig. 6). The gene expression of NEAT1 was compared by the molecular markers in the IDH-WT GBM RNAseq data. [file 12967_2020_2647_MOESM10_ESM.pdf]

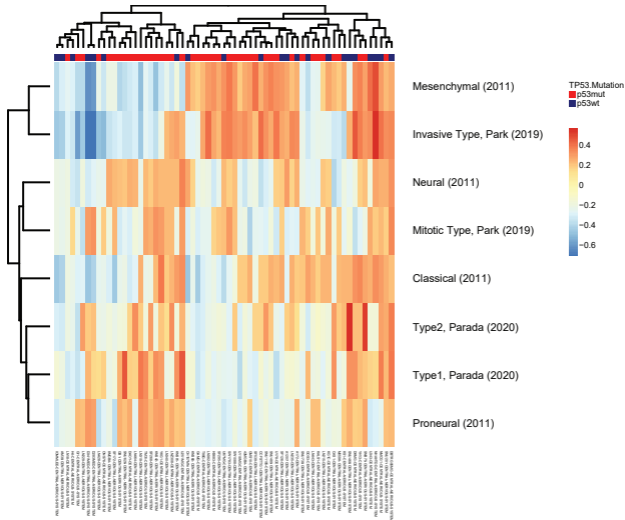

Supplement: Supplementary file 11 — Additional file 11. Subtypes of the CNS related tumor cells in the CCLE database (Related to the Fig. 4). The RNAseq data of CCLE was downloaded and analyzed by the same method for the subtype analysis (Related to Fig. 4c). Both group of TP53 mutation status cells were classified to mesenchymal (or invasive) types [1, 81]. [file 12967_2020_2647_MOESM11_ESM.pdf]

**a**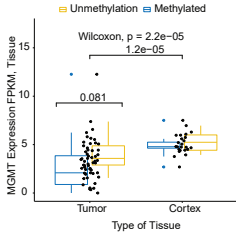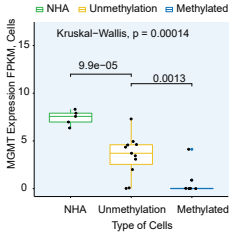**b**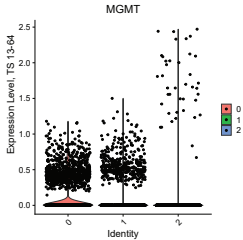

Supplement: Supplementary file 14 — Additional file 14. MGMT gene expression from the tissue, GBM TSs, and TS13-64 (Related to the Fig. 3). a. Gene expression by MGMT promoter methylation status in the GBM (n = 58; Unmethylated samples, n = 35; Methylated Samples, n = 23) and its associated control cortex tissue (n=24). GBM TSs is displayed in the right panel (Unmethylated TS n = 12, Methylated TS n = 11). [file 12967_2020_2647_MOESM14_ESM.pdf]

All TP53 WT TS (N=4)

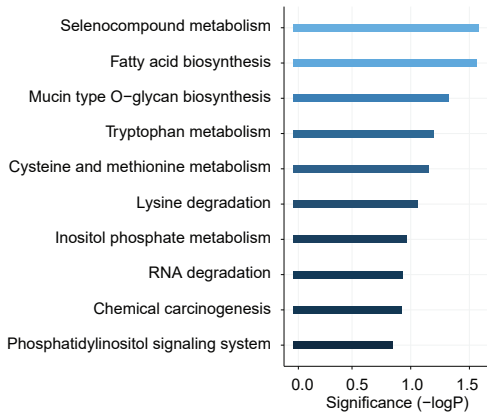

All TP53 Mutant TS (N=16)

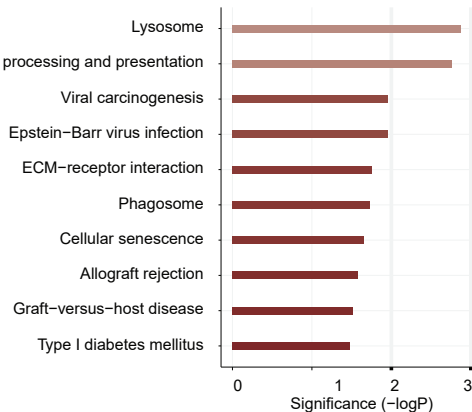

Supplement: Supplementary file 15 — Additional file 15. KEGG analysis on the GBM TSs by the mutation status of TP53 (Related to the Fig. 4). Each gene of GBM TSs (not excluding TS20-24 and 20-09) were calculated for the gene set enrichment analysis. The highly enriched gene lists were examined with the KEGG database. [file 12967_2020_2647_MOESM15_ESM.pdf]
