# Supplementary material for: Co-expression of cancer driver genes: IDH-wildtype glioblastoma-derived tumorspheres
Source: J Transl Med. 2020 Dec 14;18:482. doi: 10.1186/s12967-020-02647-8 (PMC7734785; doi:10.1186/s12967-020-02647-8)
Supplement: Supplementary file 7 — Additional file 7. Detailed description of the methods. [file 12967_2020_2647_MOESM7_ESM.docx]

## Transcriptome data analysis

The individual samples were pooled and sequenced on the Illumina NovaSeq6000 with 150 bp paired-end by following the manufacturer's protocols (Average read depth = 11). After filtering the fastq files using trimmomatic (v. 0.38) with the parameters of ‘TruSeq3-PE-2.fa:2:30:10 and MINLEN:50’ [1]. The filtered reads were mapped to the human reference genome (GRCh38.p5) using the hisat2 (2.1.0) followed by stringtie (2.0.6) with the transcript merging step, and ballgown (2.18.0) [2, 3]. Individual transcripts were reannotated using the UCSC genome browser [4].

## Mutation calling

We examined the mutational profiles of TS13-64-derived cells, a tissue sample of TS13-64, and GSC11-derived cells (which did not have a matched clinical sample for inclusion in RNAseq) with an alternative method using the RNAseq BAM file. The GenomeAnalysisToolkit protocol was used to call mutations [5]. Duplicated reads were marked with the Picard and Mutect2 algorithms (dbSNP b153 was included as a germ-line resource and no callable depth was applied), followed by filtering with the reference genome (GRCh38.p5). Protein coding mutations were filtered using the GBM 3D hotspot database  [6].

## Single cell RNAsequencing

Briefly, along with the reverse transcription master mix, a dissociated GBM TS (TS 13-64) suspension was loaded onto 10x Genomics Single Cell 30 Chips. During this step, cells were partitioned into the GEMs along with gel beads coated with oligonucleotides. These oligonucleotides enable mRNA capture inside the droplets by 30 bp oligo-dT after cell lysis and provide barcodes to index cells (16 bp) as well as transcripts (10 bp UMI). Following reverse transcription, cDNAs with both barcodes were amplified, and a library was constructed using the Single Cell 30 Reagent Kit (v2 chemistry) for each sample. The resulting libraries were sequenced on an Illumina NovaSeq 6000 System in a 2 × 150 bp paired-end mode.

Sample demultiplexing, barcode processing and UMI counting were performed by using the official 10x Genomics pipeline Cell Ranger (v3.1.0, https://support.10xgenomics.com). Briefly, raw base call files generated by Illumina sequencers were demultiplexed into reads in FASTQ format using the bcl2fastq developed by Illumina (https://github.com/brwnj/bcl2fastq). The raw reads were trimmed from the 3’ end to get the recommended number of cycles for read pairs (Read1: 26 bp; Read2: 98 bp). The reads of each library were then processed separately using the “cellranger count” pipeline to generate a gene-barcode matrix for each library. During this step, the reads were aligned to a human reference genome (version: hg19). Cell barcodes and UMIs associated with the aligned reads were subjected to correction and filtering.

## Gene set enrichment analysis (GSEA)

The gene sets of gliomasphere invasion was downloaded from mSigDB [7, 8]. We extracted invasion (or epithelial-mesenchymal transition [EMT]) related *Homo sapiens* genes from 25 gene sets of mSigDB (Search keyword: “Epithelial” AND “Mesenchymal” AND “Transition”, Aigner ZEB1 targets, Begum targets of PAX3 FOXO1 fusion and PAX3, Begum targets of PAX3 FOXO1 fusion DN, Begum targets of PAX3 FOXO1 fusion UP, Biocarta MTA3 pathway, EMT, Go cardiac EMT, Go epithelial mesenchymal cell signaling, Go EMT, Go EMT involved in endocardial cushion formation, Go mesenchymal epithelial cell signaling, Go mesenchymal to epithelial transition, Go mesenchymal to epithelial transition involved in metanephros morphogenesis, Go negative regulation of EMT, Go positive regulation of EMT, Go positive regulation of EMT involved in endocardial cushion formation, Go regulation of cardiac EMT, Go regulation of EMT, Go regulation of EMT involved in endocardial cushion formation, Go regulation of mesenchymal to epithelial transition involved in metanephros morphogenesis, Mishra carcinoma associated fibroblast DN, Mishra carcinoma associated fibroblast UP, Sarrio epithelial mesenchymal transition DN, Sarrio epithelial mesenchymal transition UP, Zhang targets of EWSR1 FLI1 fusion) [8].

1. Bolger AM, Lohse M, Usadel B. Trimmomatic: a flexible trimmer for Illumina sequence data. Bioinformatics. 2014;30(15):2114-20.

2. Pertea M, Kim D, Pertea GM, Leek JT, Salzberg SL. Transcript-level expression analysis of RNA-seq experiments with HISAT, StringTie and Ballgown. Nat Protoc. 2016;11:1650.

3. Kim D, Paggi JM, Park C, Bennett C, Salzberg SL. Graph-based genome alignment and genotyping with HISAT2 and HISAT-genotype. Nat Biotechnol. 2019;37(8):907-15.

4. Kent WJ, Sugnet CW, Furey TS, Roskin KM, Pringle TH, Zahler AM, et al. The human genome browser at UCSC. Genome Res. 2002;12(6):996-1006.

5. Van der Auwera GA, Carneiro MO, Hartl C, Poplin R, Del Angel G, Levy-Moonshine A, et al. From FastQ data to high confidence variant calls: the Genome Analysis Toolkit best practices pipeline. Curr Protoc Bioinformatics. 2013;43(1110):11.0.1-.0.33.

6. Gao J, Chang MT, Johnsen HC, Gao SP, Sylvester BE, Sumer SO, et al. 3D clusters of somatic mutations in cancer reveal numerous rare mutations as functional targets. Genome Med. 2017;9(1):4.

7. Günther HS, Schmidt NO, Phillips HS, Kemming D, Kharbanda S, Soriano R, et al. Glioblastoma-derived stem cell-enriched cultures form distinct subgroups according to molecular and phenotypic criteria. Oncogene. 2008;27(20):2897-909.

8. Subramanian A, Tamayo P, Mootha VK, Mukherjee S, Ebert BL, Gillette MA, et al. Gene set enrichment analysis: A knowledge-based approach for interpreting genome-wide expression profiles. Proc Natl Acad Sci U S A. 2005;102(43):15545-50.
