# Supplementary material for: Co-expression of cancer driver genes: IDH-wildtype glioblastoma-derived tumorspheres
Source: J Transl Med. 2020 Dec 14;18:482. doi: 10.1186/s12967-020-02647-8 (PMC7734785; doi:10.1186/s12967-020-02647-8)
Supplement: Supplementary file 13 — Additional file 13. GBM oncogene mutation profiles. The mutation profiles of the two GBM TSs (TS13-64, GSC11). [file 12967_2020_2647_MOESM13_ESM.docx]

**Additional File 2.**

**Supplementary Table 2.** GBM oncogene mutation profile.

| Gene | Transcripts | GBM Tissue 13-64 | TS1364-1 | TS1364-2 | TS1364-3 | GSC11-1 | GSC11-2 | GSC11-3 |
| --- | --- | --- | --- | --- | --- | --- | --- | --- |
| *TP53* | ENST00000269305 | c.404G>C | c.404G>C | c.404G>C | c.404G>C |  |  | c.436T>A |
| *TP53BP1* | ENST00000263801 | c.4569C>T | c.4569C>T | c.4569C>T | c.4569C>T |  |  |  |
| *DPP6* | ENST00000377770 | c.945C>T | c.945C>T | c.945C>T | c.945C>T |  |  |  |
|  | ENST00000377770 | c.723A>G |  |  |  |  |  |  |
|  | ENST00000377770 | c.1862_1863  delAC |  |  |  |  |  |  |
|  | ENST00000377770 | c.1865A>G |  |  |  |  |  |  |
|  | ENST00000377770 | c.2215C>T |  |  |  |  |  |  |
| *CUL5* | ENST00000393094 | c.1580A>G |  |  |  |  |  |  |
| *TBK1* | ENST00000331710 | c.1322A>G |  |  |  |  |  |  |
| *CDK8* | ENST00000381527 | c.791A>G |  |  |  |  |  |  |
| *KDR* | ENST00000618562 | c.588C>A |  |  |  |  |  |  |
| *EGFR* | ENST00000342916.7 | c.1887delA |  |  |  |  |  |  |
|  | ENST00000275493 | c.2549A>G |  |  |  |  |  |  |
| *AOC1* | ENST00000416793.6 | c.17G>A |  |  |  |  |  |  |
|  | ENST00000360937 | c.1155A>T |  |  |  |  |  |  |
| *BCOR* | ENST00000441294.1 | c.1447C>T |  |  |  |  |  |  |

The list of GBM oncogene was derived from 3D hotspot database [1]. The table is displayed base on the high impact mutation profile of original IDH-wildtype GBM tumor of 13-64 patient [2].

**TS:** Tumorsphere, **GSC11:** glioblastoma stem cell 11

1. Gao J, Chang MT, Johnsen HC, Gao SP, Sylvester BE, Sumer SO, et al. 3D clusters of somatic mutations in cancer reveal numerous rare mutations as functional targets. Genome Med. 2017;9(1):4.

2. den Dunnen JT, Dalgleish R, Maglott DR, Hart RK, Greenblatt MS, McGowan-Jordan J, et al. HGVS Recommendations for the Description of Sequence Variants: 2016 Update. Hum Mutat. 2016;37(6):564-9.
