# Supplementary material for: Co-expression of cancer driver genes: IDH-wildtype glioblastoma-derived tumorspheres
Source: J Transl Med. 2020 Dec 14;18:482. doi: 10.1186/s12967-020-02647-8 (PMC7734785; doi:10.1186/s12967-020-02647-8)
Supplement: Supplementary file 5 — Additional file 5. TS gene expression heatmap of the glioma sphere gene sets (Related to the Fig. 4). a. Glioma sphere downregulated genes. b. Glioma sphere upregulated genes [65]. TS: Tumorsphere. [file 12967_2020_2647_MOESM5_ESM.pdf]

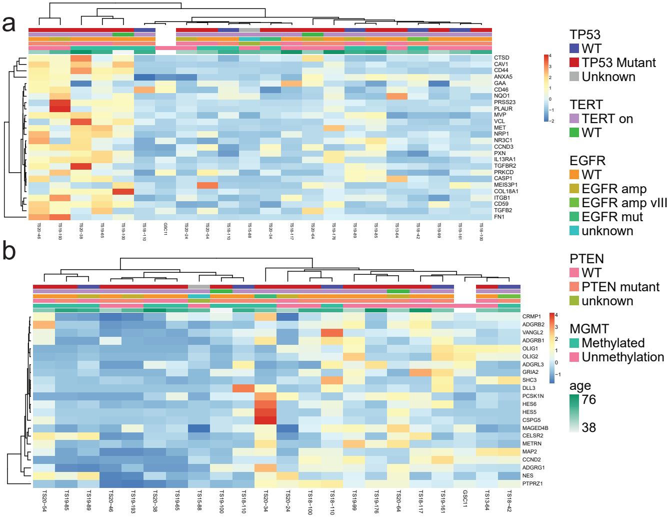

a
b
CTSD
CAV1
CD44
ANXA5
GAA
CD46
NQO1
PRSS23
PLAUR
MVP
VCL
MET
NRP1
NR3C1
CCND3
PXN
IL13RA1
TGFBR2
PRKCD
CASP1
MEIS3P1
COL18A1
ITGB1
CD59
TGFB2
FN1
CRMP1
ADGRB2
VANGL2
ADGRB1
OLIG1
OLIG2
ADGRL3
GRIA2
SHC3
DLL3
PCSK1N
HES6
HES5
CSPG5
MAGED4B
CELSR2
METRN
MAP2
CCND2
ADGRG1
NES
PTPRZ1
TS20-54
TS19-85
TS19-89
TS20-46
TS19-193
TS20-38
TS19-65
TS15-88
TS19-100
TS18-110
TS20-34
TS20-24
TS18-100
TS18-110
TS19-99
TS19-176
TS20-64
TS18-117
TS19-161
GSC11
TS13-64
TS18-42
TP53
WT
TP53 Mutant
Unknown
TERT
TERT on
WT
EGFR
WT
EGFR amp
EGFR amp vIII
EGFR mut
unknown
PTEN
WT
PTEN mutant
unknown
MGMT
Methylated
Unmethylation
age
76
38
